# Terminal Stage of Esophageal Achalasia Combined With Adenocarcinoma After a Previous Open Myotomy

**DOI:** 10.7759/cureus.76833

**Published:** 2025-01-02

**Authors:** Georgi Yankov, Magdalena Alexieva, Zaharinka Makshutova, Stefka Yankova, Evgeni V Mekov

**Affiliations:** 1 Department of Thoracic Surgery, University Hospital “St. Ivan Rilski”, Medical University of Sofia, Sofia, BGR; 2 Department of Thoracic Surgery, Medical University of Sofia, Sofia, BGR; 3 Department of Nephrology and Dialysis, University Children's Hospital “Prof. Ivan Mitev”, Sofia, BGR; 4 Department of Respiratory Diseases, Medical University of Sofia, Sofia, BGR

**Keywords:** achalasia, adenocarcinoma, diagnostics, esophagectomy, gastroesophagoplasty, treatment

## Abstract

Achalasia is a rare, chronic disorder of esophageal motility characterized by the lower esophageal sphincter's inability to relax and a lack of normal esophageal peristalsis. We present a 70-year-old man with longstanding achalasia, a previous open myotomy, and adenocarcinoma in the lower third of the esophagus. A subtotal esophageal resection, gastroesophagoplasty by* *McKeown, and pyloroplasty by Heineke-Mikulicz were performed.

## Introduction

Achalasia is a rare, chronic disorder of esophageal motility characterized by the lower esophageal sphincter (LES) 's inability to relax and a lack of normal esophageal peristalsis. This condition results from the degeneration of the neurons in the myenteric plexus, leading to dysphagia, regurgitation, and, in advanced cases, weight loss and malnutrition [1].

Diagnosis of achalasia relies on a combination of clinical evaluation and specialized investigations. High-resolution manometry is the gold standard, demonstrating the hallmark features of impaired LES relaxation and aperistalsis [2]. Esophagography, particularly with a barium swallow, can reveal a "bird-beak" appearance of the LES and a dilated, tortuous esophagus [2].

Two primary forms of achalasia are recognized: primary (idiopathic) and secondary (pseudoachalasia). Primary achalasia develops without an apparent cause, whereas secondary achalasia arises due to an underlying condition, such as malignancy or Chagas disease. Patients with achalasia are at increased risk of esophageal carcinoma for both squamous and adenocarcinoma [3,4].

Therapeutic approaches for achalasia include endoscopic, pharmacological, and surgical methods aimed at relieving symptoms and restoring esophageal function. Among these, surgical intervention, such as Heller myotomy, is a cornerstone treatment, particularly for advanced cases [5]. However, even after surgical treatment, some patients may experience recurrent symptoms or develop complications, including malignancy. In such scenarios, esophagectomy emerges as the definitive therapeutic option.

We present a 70-year-old man with longstanding achalasia, previous open myotomy, and adenocarcinoma in the lower third of the esophagus. A subtotal esophageal resection, gastroesophagoplasty by McKeown, and pyloroplasty by Heineke-Mikulicz were performed. This report highlights the need for multidisciplinary approaches and demonstrates the efficacy of esophagectomy in treating advanced cases with malignancy.

## Case presentation

A 70-year-old man was admitted to the thoracic surgery department with complaints of dysphagia for solid and liquid foods with periods of spontaneous improvement, periodic vomiting, and weight reduction of 10 kg in the last month. Thirty years ago, the patient underwent a myotomy and diaphragmо-fundopexy through an abdominal approach in another institution, but the effect of this intervention was unsatisfactory. After this intervention, several pneumatic dilations were performed with a temporary effect.

**Figure 1:**
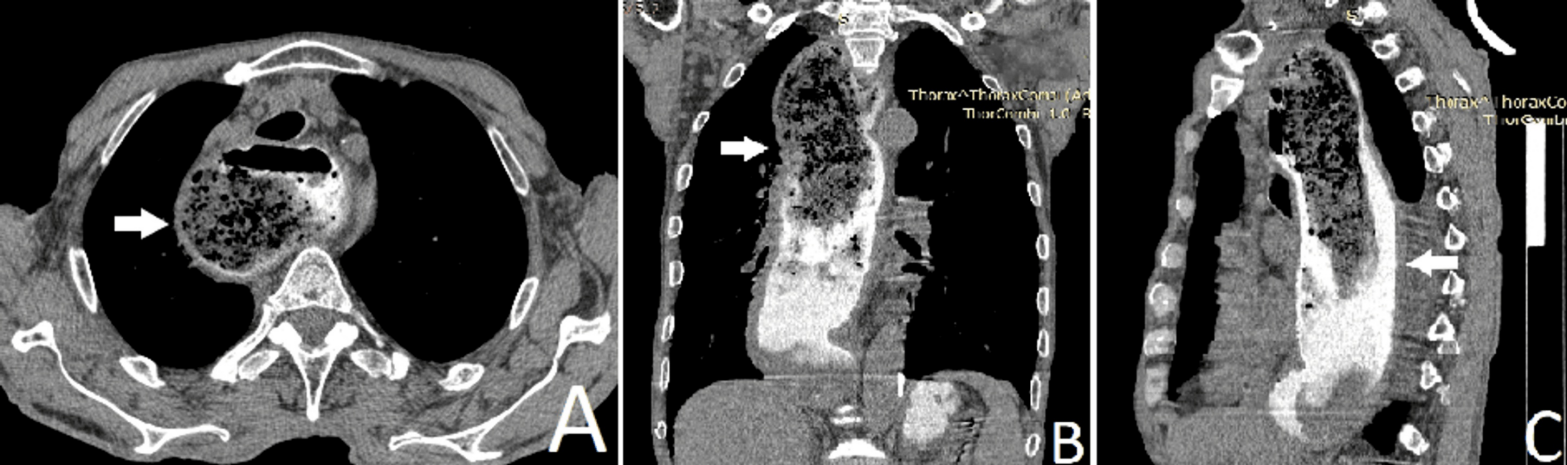
Аxial (A), coronary (B), and sagittal (C) CT views of the thoracic esophagus (the arrows show the dilated and tortuous esophagus) CT: computed tomography

Fiberoptic esophagogastroscopy (FEGS) shows a greatly dilated, tortuous esophagus filled with fluid and stagnant food. Absence of peristalsis and mucous membrane with whitish plaques, hyperemic and edematous, was established. А forceps biopsy was taken from the distal third of the esophagus, but tumor cells were not detected on the final histological examination. An endoscopic diagnosis was achalasia of the esophagus and erosive gastritis. A computed tomography (CT) study was performed, and it showed centrilobular emphysema. An intrathoracic esophagus was presented with a dilated lumen in the axial plane, filled with food debris and oral contrast material (Figure 1). The wall is circularly thickened up to 8 mm. In the distal pole and the gastroesophageal junction, the lumen is not visualized. The stomach wall in the fundus part is thickened, and numerous calcium-dense stones are visualized in the gallbladder lumen and the left kidney. There were CT data for at least two enlarged lymph nodes along the small curvature of the stomach up to 12 mm.

The decision to proceed with esophagectomy was driven by the combination of the patient’s longstanding history of achalasia, the ineffectiveness of prior interventions, and imaging findings suggestive of malignancy. The diagnostic process emphasized a systematic approach to clinical evaluation, endoscopic assessment, and CT imaging.

**Figure 2:**
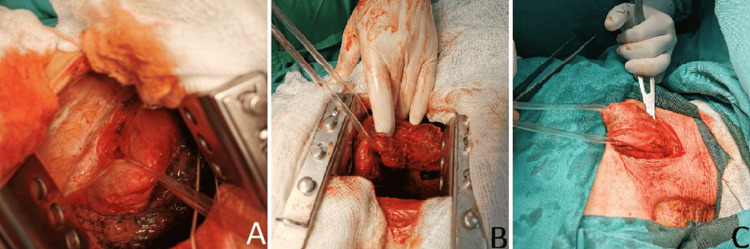
Intraoperative view. Dissection and mobilization of the (A,B) thoracic esophagus and (C) neck esophagus

A subtotal esophageal resection and gastroesophagoplasty by Ivor-Lewis and McKeown were performed. A posterolateral right thoracotomy was accomplished along the course of the fifth intercostal space. A highly dilated, tortuous, and thick-walled thoracic megaesophagus with multiple vessels located subadventitially and severe periesophageal adhesions were found (Figure 2). The mediastinal pleura was incised from the diaphragm to the upper thoracic aperture, and the azygos vein was divided. Enlarged periesophageal, paratracheal, and carinal lymph nodes, exhibiting predominantly inflammatory changes, were excised. The thoracic esophagus was mobilized from the esophageal hiatus to the upper thoracic aperture. Numerous dilated and hypertrophied arterial branches originating from the thoracic aorta, along with accompanying venous branches, were ligated and severed. The thoracic duct was also ligated in the area above the hiatus. Simultaneously, a left vertical neck incision and upper median laparotomy were performed. The cervical esophagus was dissected, and a subtotal resection was completed approximately 2 cm below the hypopharynx (Figure 2). In the abdominal cavity, extensive adhesions were observed between the anterior stomach wall and an enlarged, slightly thickened liver. These adhesions were sharply divided. Further adhesions were encountered around the abdominal esophagus and the severely narrowed hiatus due to the prior intervention.

**Figure 3:**
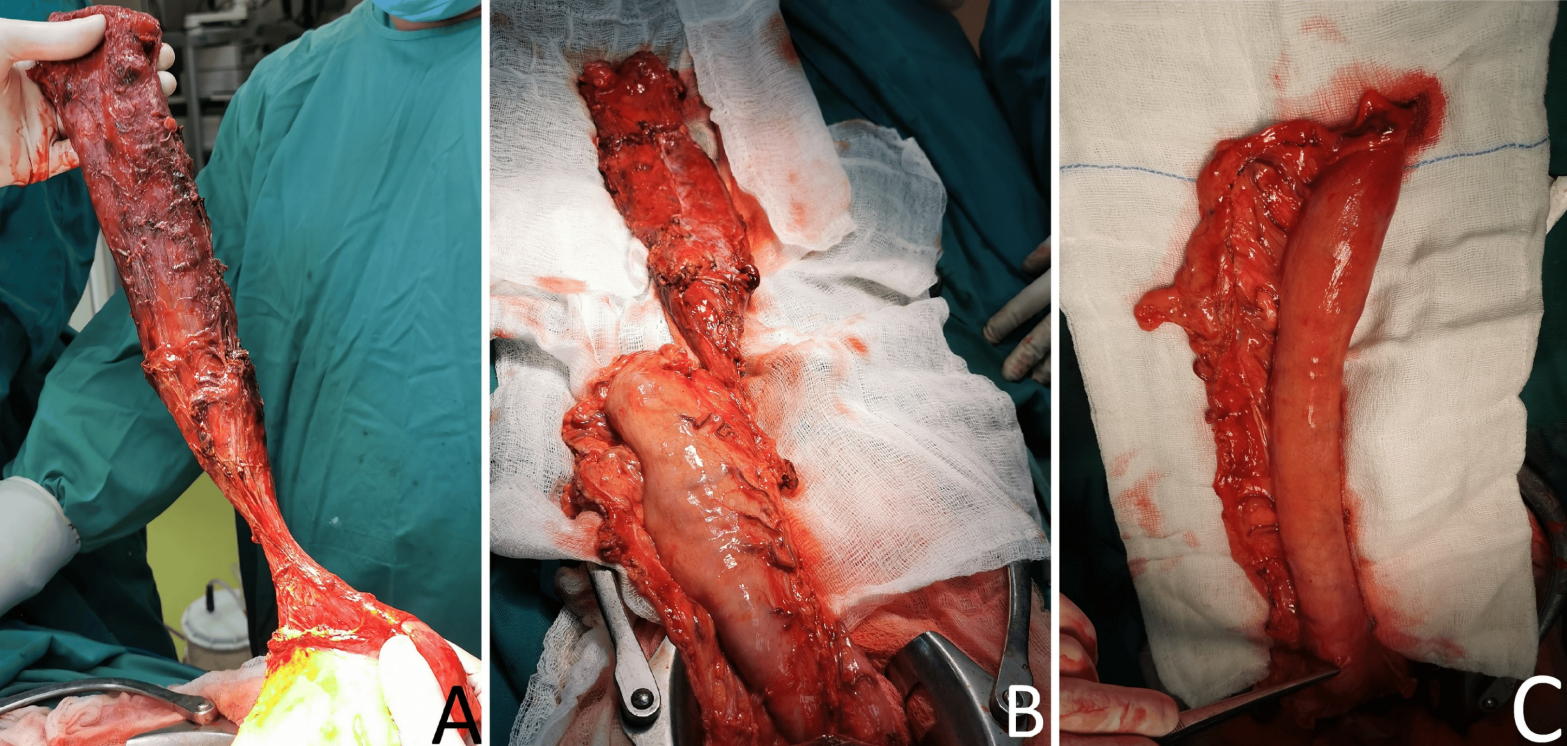
(A,B) A skeletonized esophagus and a stomach. (C) A neoesophagus created by the greater curvature

Lymph nodes in the region of the left gastric artery, celiac trunk, common hepatic artery, and splenic artery were mildly enlarged and thickened; these, along with nodes along the lesser curvature of the stomach, were excised. The lesser curvature of the stomach was skeletonized and mobilized, preserving the right gastroepiploic artery as the feeding vessel. A gastric tube was created (Figure 3), and the duodenum was mobilized by the Kocher maneuver. Heineke-Mikulicz pyloroplasty was subsequently performed. Additionally, due to chronic calculous cholecystitis with dense and thickened gallbladder walls, a cholecystectomy was conducted.

**Figure 4:**
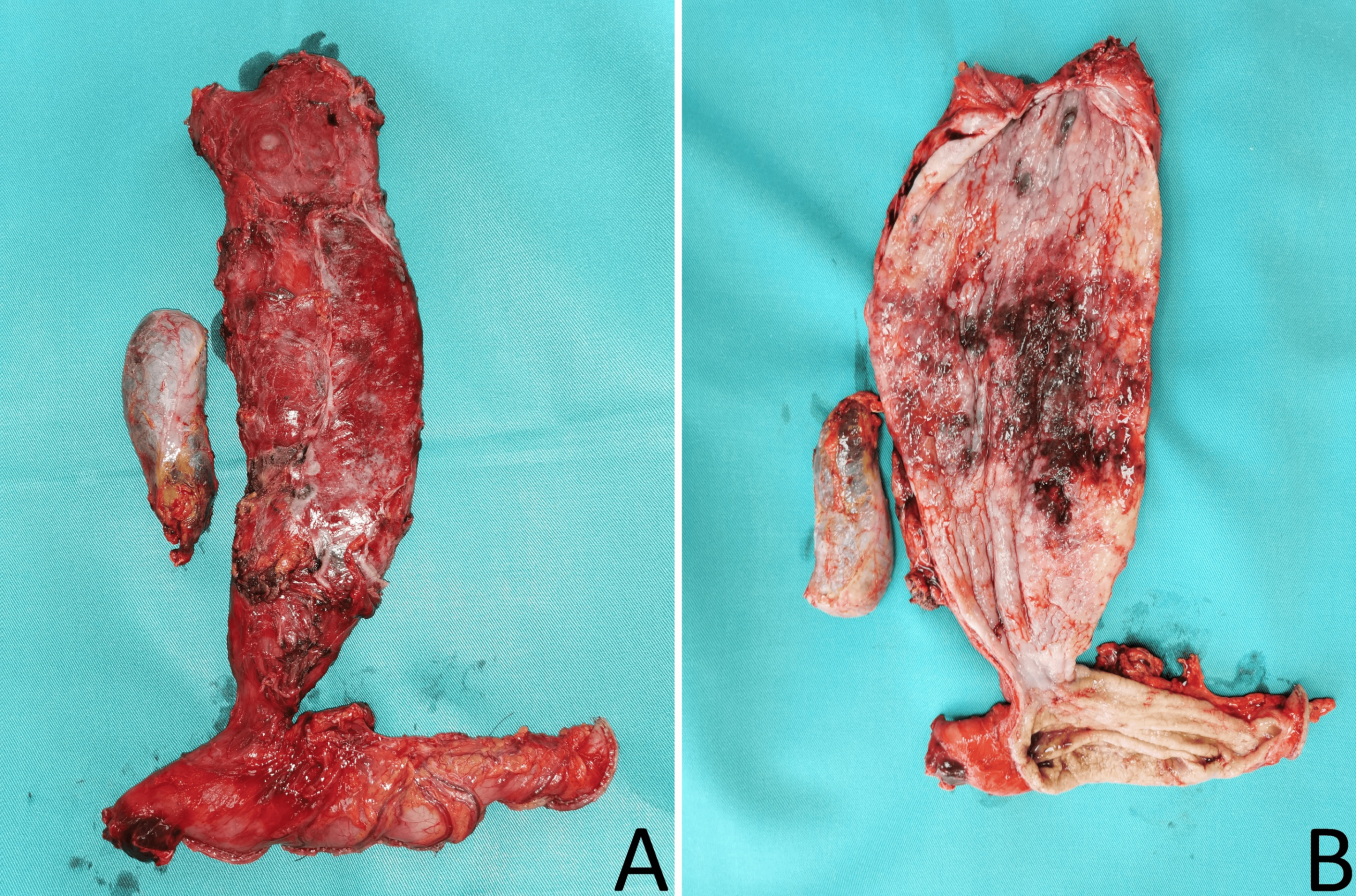
Macroscopic view of the resected specimen. (A) Resected esophagus, upper part of the stomach, and gallbladder. (B) Dissected view of the esophagus, stomach, and gallbladder

The digestive tract continuity was restored using a McKeown cervical terminolateral esophagogastrostomy. The macroscopic specimens, including the resected esophagus, a portion of the lesser curvature of the stomach, and the gallbladder, are shown in Figure 4.

The histological result shows esophagus wall (lower third) with Barrett's mucosa and transition to intramucosal adenocarcinoma with lamina propria infiltration; shows the absence of lymphovascular invasion; emphasized absence of ganglion cells in the plexus myentericus (in the middle part of the esophagus) and replacement with connective tissue and muscle hypertrophy; shows that the squamous mucosa is markedly hypertrophic with papillomatosis, shows reactive changes and basal cell hyperplasia resembling gastroesophageal reflux disease; and subepithelially expressed lymphoplasmacytic inflammatory infiltrate with the formation of lymphatic follicles with germinal centers. A total of 23 lymph nodes were examined, all without metastases. The gallbladder showed evidence of chronic calculous cholecystitis, characterized by dense and thickened walls. The resection lines are free of tumor infiltration (pT1a N0 R0). The postoperative period was uneventful. The patient was discharged on the 10th postoperative day. Two years later, he is in excellent overall condition.

## Discussion

Achalasia is a primary esophageal motility disease with an unrecognized cause, marked by esophageal aperistalsis and constricted LES, manifesting with dysphagia and regurgitation [6]. This is a rare disorder with a frequency of nearly one in 100,000 people, with no gender or race predilection [7], as it usually affects patients between 40 and 60 years [2]. Two types of achalasia are known: primary (idiopathic) and secondary (pseudoachalasia).

We present a 70-year-old man with primary achalasia and an open myotomy performed 30 years ago. The effect of this intervention was not good, and it was difficult for the patient to eat again. Dilations were periodically performed with a temporary effect.

We performed an esophagectomy because of the previous open myotomy and the long-term course of the disease. Expectations of massive adhesions in the peritoneal cavity with altered anatomy, as well as severe periesophageal adhesions, were confirmed during the intervention. We decided to start with open surgery due to an unclear preoperative diagnosis, suspicion of malignancy, and expectation of severe periesophageal adhesions due to the long duration of the disease. The operation was extremely difficult because of the massive adhesions and the multiple arterial branches arising directly from the thoracic aorta and accompanying venous branches, each of which had to be treated separately. A postoperative period was uneventful, with excellent late results.

Endoscopic evaluation is advocated for the exclusion of pseudoachalasia caused by malignancy, peptic stricture from acid reflux, esophageal webs and rings, esophageal inflammation, and eosinophilic esophagitis [2]. Risk factors for malignant pseudoachalasia are age >55 years, weight loss >10 kg, duration of symptoms <12 months, and difficulty in LES passing with an endoscope [8]. In the presented case, the FEGS revealed a dilated esophagus filled with nutrients, but malignancy was not proven preoperatively, probably because of the food-filled esophagus and poor visibility.

CT scan or barium swallow study are more helpful and reliable methods than upper endoscopy for achalasia screening [9]. CT features are the thickness of the esophageal wall at the body and distal esophageal segment (DES), nodular/lobulated appearance of DES (with a predominant appearance in subtype III), ground-glass lung opacities, tracheal/carinal compression, and left atrial compression [6]. CT scanning is offered in patients 50 years old with a new-onset and rapidly developing dysphagia [10]. Preoperative imaging plays a crucial role in planning surgical interventions for advanced achalasia, particularly when complicated by malignancy. In this case, CT provided critical insights into the anatomical challenges that would be encountered during surgery. In our case, the performed CT found a dilated and tortuous thoracic esophagus in its entire course. The CT findings and the long history of the achalasia are pointing to malignancy.

Achalasia is a premalignant disorder, as the risk of squamous cell carcinoma development is from 1 to 140 times that of the normal population, and in addition, it should also predispose to Barrett's metaplasia and adenocarcinoma evolution [11]. Barrett's esophagus in achalasia is usually the result of gastroesophageal reflux after esophagomyotomy, as in 73% of cases in one analysis [12]. There is an increased risk of neoplastic degeneration in achalasia patients due to severe histological esophagitis and a high Ki-67 proliferation index in the distal esophageal third [13]. Continuous chemical esophageal irritation in response to food debris and saliva should elicit chronic hyperplastic esophagitis, dysplasia, and carcinoma [5]. Adenocarcinoma may develop in 20% of the patients with achalasia and Barrett's esophagus [12].

The performance of esophagectomy in end-stage achalasia patients is a safe and effective procedure advised in case of disabling symptoms, poor quality of life, and dolichomegaesophagus refractory to multiple dilations and/or myotomies [14].

## Conclusions

Achalasia is a progressive and debilitating disease with an incompletely elucidated etiology. The combination of achalasia with adenocarcinoma after previous surgery represents a unique and challenging clinical scenario. Esophagectomy remains the method of choice for managing end-stage achalasia, particularly when complicated by malignancy, as it addresses both the functional and oncological aspects of the disease. Esophagectomy, although technically demanding, offers excellent outcomes when performed in well-selected patients. The uneventful recovery and the patient's sustained improvement two years postoperatively highlight the potential for favorable long-term results, even in complicated cases.
